# Volcanic‐Tectonic Structure of the Mount Dent Oceanic Core Complex in the Ultraslow Mid‐Cayman Spreading Center Determined From Detailed Seafloor Investigation

**DOI:** 10.1029/2018GC008032

**Published:** 2019-03-07

**Authors:** G. A. Haughton, N. W. Hayman, R. C. Searle, T. Le Bas, B. J. Murton

**Affiliations:** ^1^ School of Ocean and Earth Sciences University of Southampton Southampton UK; ^2^ Institute for Geophysics, Jackson School for Geosciences University of Texas Austin TX USA; ^3^ Department of Earth Sciences Durham University Durham UK; ^4^ National Oceanography Center Southampton UK

**Keywords:** Oceanic Core Complex, detachment fault, ultraslow spreading ridge, mid‐ocean ridge

## Abstract

The flanks of the ultraslow‐spreading Mid‐Cayman Spreading Center (MCSC) are characterized by domal massifs or oceanic core complexes (OCCs). The most prominent of these, Mount Dent, comprises lower‐crustal and upper‐mantle lithologies and hosts the Von Damm vent field ~12 km west of the axial deep. Here, presented autonomous underwater vehicle‐derived swath sonar (multibeam) mapping and deep‐towed side‐scan sonar imagery lead to our interpretation that: (i) slip along the OCC‐bounding detachment fault is ceasing, (ii) the termination zone, where detachment fault meets the hanging wall, is disintegrating, (iii) the domed surface of the OCC is cut by steep north‐south extensional faulting, and (iv) the breakaway zone is cut by outward facing faults. The Von Damm vent field and dispersed pockmarks on the OCC's south flank further suggest that hydrothermal fluid flow is pervasive within the faulted OCC. On the axial floor of the MCSC, bright acoustic backscatter and multibeam bathymetry reveal: (v) a volcanic detachment hanging wall, (vi) a major fault rifting the southern flank of Mount Dent, and (vii) a young axial volcanic ridge intersecting its northern flank. These observations are described by a conceptual model wherein detachment faulting and OCC exhumation are ceasing during an increase in magmatic intrusion, brittle deformation, and hydrothermal circulation within the OCC. Together, this high‐resolution view of the MCSC provides an instructive example of how OCCs, formed within an overall melt‐starved ultraslow spreading center, can undergo magmatism, hydrothermal activity, and faulting in much the same way as expected in magmatically more robust slow‐spreading centers elsewhere.

## Key Points

- The Mid‐Cayman Spreading Center is an example of how magmatism can drive cycles of ocean core complex development even at ultraslow rates
- Decreasing detachment fault slip is marked by crosscutting higher angle faults and patterns of mass wasting and sedimentation
- The surface expression of hydrothermal fluid flow in vents and pockmarks is linked with the mechanical evolution of oceanic core complexes

## 1 Introduction

Mid‐ocean ridges accommodate seafloor spreading via a combination of magmatic and tectonic processes (Cann, 1968; Macdonald & Luyendyk, 1977; Mutter & Karson, 1992; Shaw & Lin, 1993; Smith & Cann, 1990; Sykes, 1967). Where the magmatic component of seafloor spreading is low and tectonic extension is high, the oceanic basement may be characterized by large‐offset detachment (normal) faults that dip shallowly at the surface, yet accommodate significant seafloor spreading resulting in the exhumation of lower‐crustal and upper‐mantle rocks at the seafloor to form oceanic core complexes (OCCs; Cann et al., 1997; Cannat, 1993; Cannat et al., 2006; Escartin et al., 2008; Ildefonse et al., 2007; Karson & Dick, 1983; Schouten et al., 2010; Tucholke & Lin, 1994; Tucholke et al., 1998, 2008). Indeed, geodynamic modeling has found that OCCs appear to form in environments where magma, intruded into the brittle lithosphere, accommodates between 30% and 50% of the total plate separation (Behn & Ito, 2008; Buck et al., 2005).

Observations along the slow‐spreading Mid‐Atlantic Ridge (MAR), combined with geodynamic modeling, suggest that OCCs evolve via a “rolling hinge,” wherein the OCC detachment fault initiates at a higher angle and then, as a result of flexure and exhumation of the lower crust and/or upper mantle, is back‐tilted to emerge as a domal footwall (deMartin et al., 2007; Garces & Gee, 2007; Lavier et al., 1999; Morris et al., 2009). Following this exhumation, at some point OCCs are rendered inactive and are passively transported off axis. Understanding this “life cycle” (cf., MacLeod et al., 2009) thus hinges on understanding which processes dominate the late‐stage OCC evolution (e.g., Reston et al., 2002). MacLeod et al. (2009) argue that for OCCs at slow‐spreading ridges the detachment fault migrates past the spreading axis resulting in magmatic intrusion into the footwall, across the detachment fault, and into the brittle hanging wall, thereby ceasing the continued exhumation of the OCC. Other models of OCC life cycles envision elevated amount of magmatic intrusion to cause mechanically favorable conditions for high‐angle faults to cut across OCCs (Olive et al., 2010; Tucholke et al., 2008). But could such magmatic controls on OCC development be important in ultraslow spreading centers that are thought to be generally magma poor (e.g., Dick et al., 2003)? Furthermore, what roles might hydrothermal activity play in OCC evolution via mechanical linkages with faulting (e.g., Hirose & Hayman, 2008) and cooling of magmatic bodies within OCCs (e.g., Canales et al., 2017)?

Here, we provide evidence that a well‐developed OCC at the ultraslow spreading Mid‐Cayman Spreading Center (MCSC), is in the process of “dying” as slip on the detachment fault ceases, magmatism intrudes the OCC's footwall, and faulting accommodates extension internal to the OCC. Variations in acoustic backscatter and microbathymetry from autonomous underwater vehicle (AUV) data, deep‐towed side‐scan sonar, and shipboard multibeam data reveal the spatial and, in some cases, temporal distribution of faulting across and along the OCC. We posit that this structural evolution is intimately linked with magmatism and hydrothermal activity, the latter expressed at the Von Damm Vent Field (VDVF; Connelly et al., 2012). At some stage in this evolution, the OCC will then be transported off axis by axial seafloor spreading, as has occurred for previous OCCs along the spreading center (Grevemeyer et al., 2018). By documenting the structural geology observed at the surface with a range of seafloor‐imaging data sets, we offer a case study in the magmatic and tectonic mechanisms underlying the cessation of OCC development along an ultraslow spreading center.

## 2 Tectonic Setting

**Figure 1:**
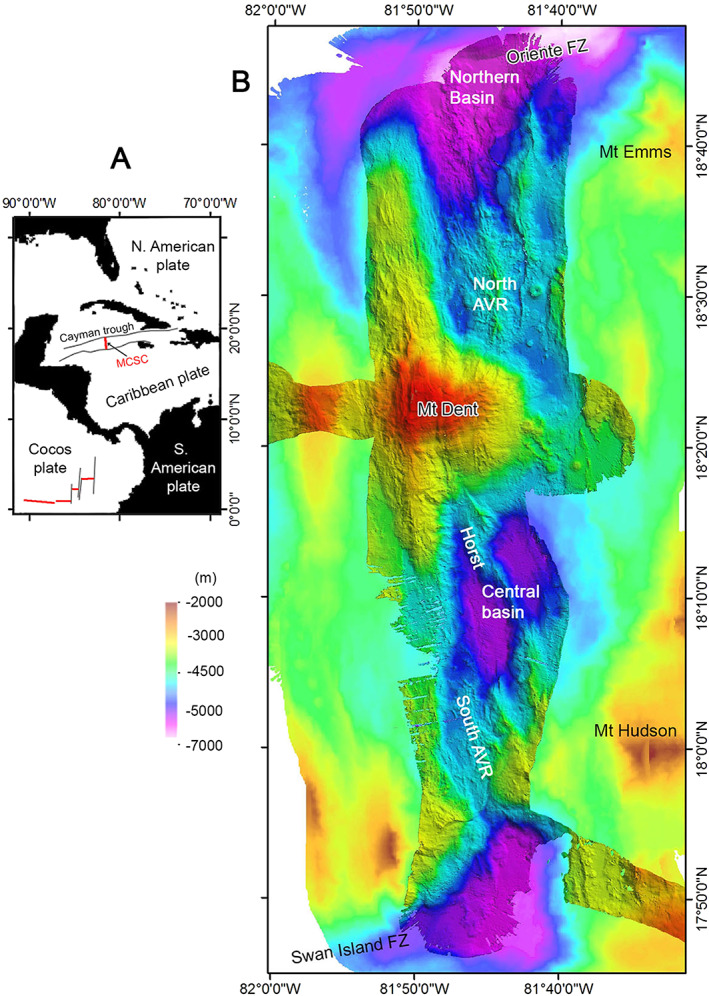
(a) Location and geological setting of the Mid‐Cayman Spreading Center (MCSC). (b) The 50‐m‐gridded ship board multibeam bathymetry of the Mid‐Cayman Spreading Center showing the location of Mount Dent and the other major morphotectonic features discussed in the text: AVR = axial volcanic ridge, FZ = fracture zone. Mount Emms and Mount Hudson were named in Cheadle et al. (2012) and are also OCCs on the inside corners of the MCSC intersection with the adjacent fracture zones.

The OCC we focus on is known as Mount Dent (Edgar et al., 1991), and it separates the northern and southern segments of the MCSC (Figure 1). The MCSC is among the world's deepest and slowest spreading centers, having an axial depth of ~5,000–7,000 m and an ultraslow full‐spreading rate of ~15 mm/year (Macdonald & Holcombe, 1978; Rosencrantz et al., 1988). At 110‐km long, the MCSC is bound by two transform faults: the Oriente Fracture Zone to the north and the Swan Island Fracture Zone to the southwest. The MCSC formed as a pull‐apart basin between these two transform faults to accommodate Caribbean‐North American plate motion and retreat of the Caribbean/Lesser Antilles arc (Leroy et al., 2000; Mann et al., 2007). Gravity analyses and seismic imaging suggest that the MCSC hosts basaltic, gabbroic, and exhumed (serpentinized) mantle peridotite, and thus has been spreading by a mixture of magmatic accretion and tectonic spreading for the last ~10 Myr (Grevemeyer et al., 2018; ten Brink et al., 2002). In fact, there is a clear record of ultraslow seafloor spreading dating back to at least ~20 Ma, if not ~49 Ma (Hayman et al., 2011; Leroy et al., 2000).

As is the case for ultraslow spreading centers worldwide (Edmonds et al., 2003; Michael et al., 2003; Sauter et al., 2004; Tao et al., 2012), the MCSC is well known to host basaltic and gabbroic rocks along with exhumed mantle rocks (Hayman et al., 2011; Stroup & Fox, 1981), as well as hydrothermal activity (Connelly et al., 2012; German et al., 2010). Yet, the deep axial depths and incompatible‐element‐enriched Mid‐Ocean Ridge Basalt (MORB) compositions are indicative of some of the lowest potential temperature and melt fractions of any mid‐ocean ridge mantle (Klein & Langmuir, 1987). Similarly, seismic data suggest that there is a wide range of crustal thicknesses in the Cayman Trough, with some areas hosting ~3–5 km of crust, significantly thinner than crustal sections on slow‐spreading centers overall, and other areas comprising only exhumed mantle (Grevemeyer et al., 2018). Some of the thicker sections (~5 km) of MCSC lower oceanic crust accreted in zones of deep partial melt and ultimately formed OCCs (Harding et al., 2017; Hayman et al., 2011). Within the northern and southern areas of the axial deep, basaltic basins overlie ongoing lower crustal accretion, though here too the crust is thin relative to global averages (van Avendonk et al., 2017). Lastly, peridotite samples from the MCSC are evidence of truly amagmatic seafloor spreading, preserving geochemical signatures that share some similarity to Gakkel Ridge and Southwest Indian Ridge mantle (Mallick et al., 2014). Thus, the MCSC is an overall melt‐poor environment relative to the global mid‐ocean ridge system, though in detail there are areas of robust magmatism. Our effort here is to better understand how OCCs evolve in such an environment.

## 3 Methodology

The data used here were collected during RRS *James Cook* cruises JC44 and JC81 in 2010 and 2013, respectively. Shipboard swath bathymetry data, acquired using a Kongsberg‐Simrad EM120 multibeam sonar operating at a frequency of 10 kHz and at a speed over the ground of 2 kts, were filtered for spikes and errors before being gridded at 50 m. Side‐scan sonar imagery was acquired from a deep‐towed 30‐kHz system (TOBI), which was deployed at an average altitude of ~300 m above the seafloor and insonified the axial zone of the MCSC. The data were corrected for vehicle altitude (slant‐range), geographic position, and speed over the seafloor before being gridded at 6 m. High‐resolution swath bathymetry and acoustic backscatter data were acquired using a 200‐kHz Kongsberg‐Simrad EM2000 multibeam sonar, fitted to the AUV Autosub6000 operating at an average altitude of 150 m above the seafloor. Subsea navigation was provided by a combination of ultrashort baseline acoustic tracking from the surface vessel, inertial navigation, and Doppler velocity logging. The AUV bathymetry and acoustic backscatter intensity were gridded at 1 m and the final grid position adjusted to match major features seen on the GPS‐navigated shipboard bathymetry maps. These data were imported into ArcGIS™ and analyzed using a combination of raster‐ and vector‐based tools. Acoustic backscatter, either from TOBI side‐scan sonar or AUV‐derived multibeam swath sonar, is shown with light gray as high amplitudes and indicates seafloor with high acoustic albedo. Bathymetric and backscatter images reveal a variety of morphologies and textures from which features are identified (Blondel & Murton, 1997; Searle et al., 2010) including: smooth high‐reflectivity areas indicative of sheet flow lavas; smooth low‐reflectivity areas indicative of soft sediment cover; high‐frequency, low‐amplitude topography with moderate to high mottled acoustic reflectivity indicative of hummocky volcanic terrain, and circular or crescent‐shaped features indicative of volcanic edifices. The intensity of acoustic backscatter varies inversely with the thickness of pelagic sediment cover.

Fault scarps are identified as linear or curvilinear marking the traces of slopes in excess of 40°. Such offsets in seafloor depth are in many places, but not everywhere, associated with higher intensity sonar backscatter depending on sediment cover. Where appropriate, slope azimuth and inclination maps for the faults were generated from 3 × 3 matrices applied as a high‐pass filter over the gridded bathymetry data and centered on each grid element. The choice of 40° slope for the fault identification probably under samples the fault population and is considered here a conservative estimate. The high‐resolution survey areas were subsequently visually surveyed and sampled using the robotic underwater vehicle (RUV), HyBIS (Murton et al., 2012) and the ROV Isis. We make reference to the ROV and RUV observations below but do not present them in any detail as our focus is the regional interpretations of the bathymetric and backscatter data sets.

## 4 Results

### 4.1 Geology of the MCSC

**Figure 2:**
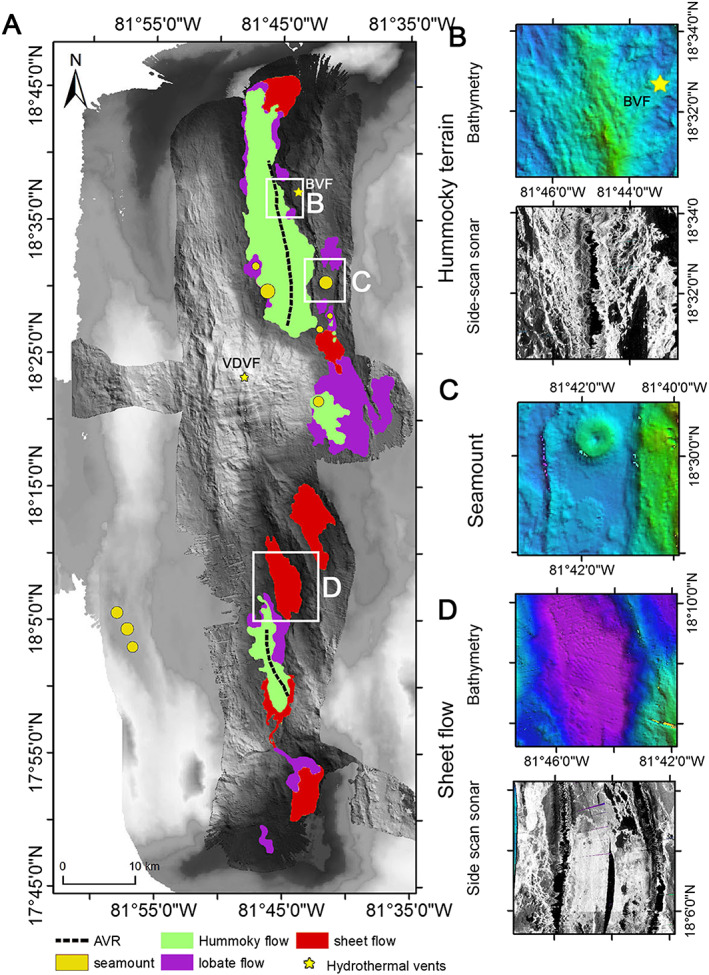
(a) Map of MCSC showing the four different volcanic terrains. BVF and VDVF are the Beebe and Von Damm Vent Fields; see Figure 1b for a colored version with bathymetric scale. Inset panels illustrate the terrain types in more detail and using side‐scan sonar to help identify each volcanic terrain type: (b) hummocky terrain, (c) seamount, (d) sheet flows.

Based on shipboard and AUV‐derived multibeam bathymetry, deep‐towed side‐scan sonar imagery (30 kHz TOBI), and near‐bottom video surveying and sampling, we can divide the MCSC into three distinct segments (Figures 1b and 2a): (1) a northern segment containing circular volcanoes and a ridge of hummocky lavas that extends into the nodal deep basin marking the intersection with the Oriente Fracture Zone, (2) a central segment dominated by the Mount Dent massif, and (3) a southern segment comprising, from north to south, several smooth floored basins, divided by a number of prominent NW‐SE trending morphological ridges and a field of hummocky and sheet flow lavas, respectively.

The northern segment is dominated by a hummocky volcanic field, identified in TOBI side‐scan sonar records as having high acoustic backscatter and a typical mottled appearance reflecting the presence of numerous small volcanic cones, as seen in other spreading centers (e.g., Searle et al., 2010). This hummocky volcanic field fills 85% of the width of the MCSC axial valley floor (Figures 2a and 2b), which is itself bounded by N‐S normal fault scarps (near 81°44′W and 81°50′W) forming the inner axial valley walls. This field includes a number of circular volcanic edifices, up to 2.5 km in diameter, some with distinct craters (Figure 2c). The center of the volcanic field contains a 9‐km‐long axial volcanic ridge (AVR) that rises up to 600 m from the valley floor. The AVR has a series of oblique ridges trending NW and NE away from its crest. One of these, on the eastern flank of the AVR, hosts an 800‐m diameter volcanic pillow‐lava mound on top of which is the deepest (5,000 m) known high‐temperature hydrothermal vent field and seafloor massive sulfide deposit, the Beebe Vent Field (BVF; Connelly et al., 2012), also known as the Piccard Vent Field (German et al., 2010; McDermott et al., 2018). The BVF comprises a series of sulfide mounds and black‐smoker chimneys venting supercritical fluids at up to 410 °C (Webber et al., 2015), with compositions that indicate a fluid‐rock reaction zone located ~3 km below the seafloor that involves both mafic and ultramafic rocks (McDermott et al., 2018; Webber et al., 2015). At the northern end of the AVR, hummocky volcanic terrain and sheet flows cover most of the floor of the 7,000‐m nodal deep basin that marks the junction between the MCSC and the Oriente Fracture Zone. The southern end of the AVR terminates abruptly against the northern flank of Mount Dent. Here, the AVR is at its most prominent, with the crest of the AVR and its hummocky lava flanks clearly visible in the bathymetry data with bright acoustic backscatter indicating it is relatively sediment free (Figure 2b).

**Figure 3:**
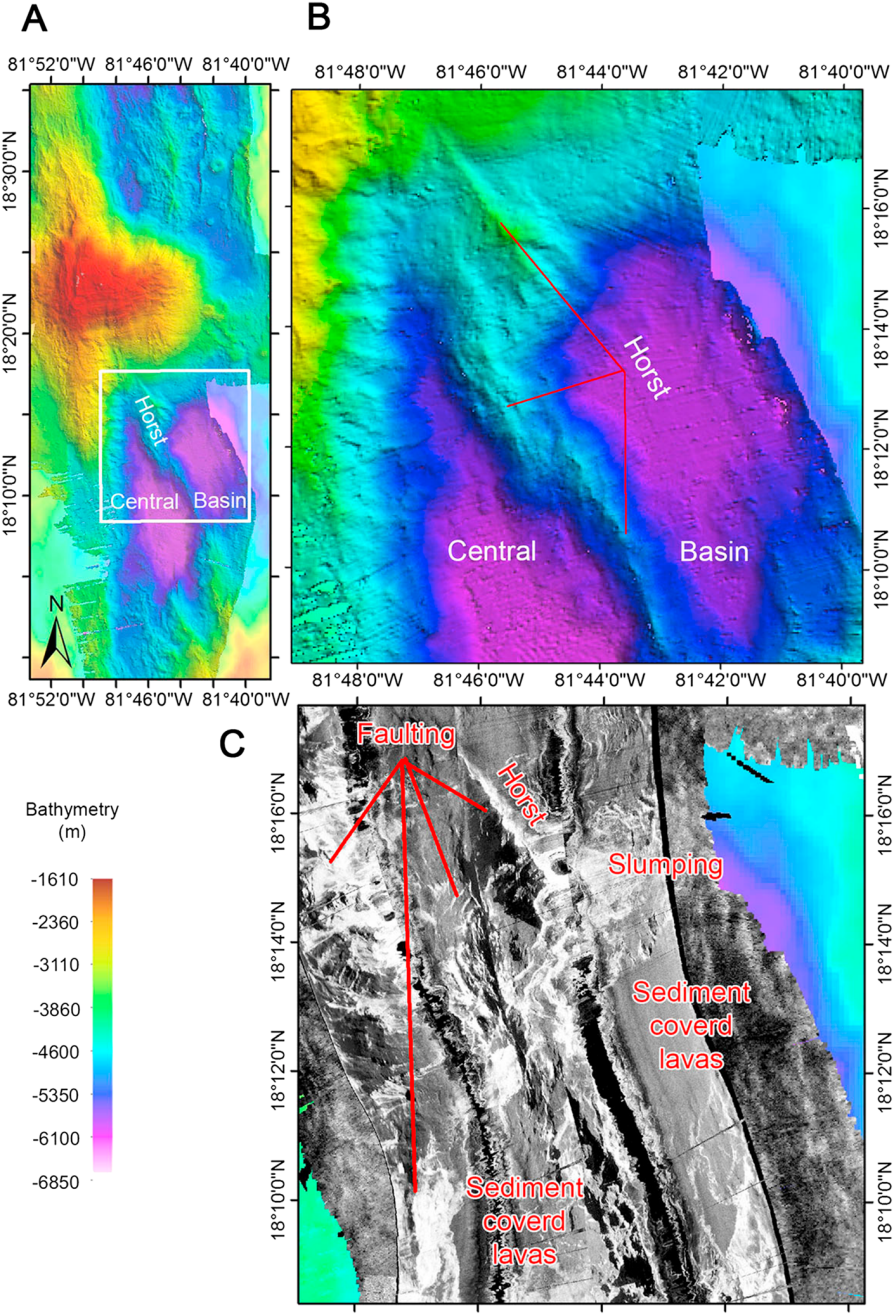
(a) Location map of the southern segment, Central Basin, and Horst. (b) Shaded relief bathymetry of the Horst with faulted regions labeled, based on high backscatter in TOBI data. (c) Side‐scan sonar image of the Central Basin and Horst. Faults appear as brightly (acoustic) backscattering curvilinear reflectors, while sheet flows are smooth with moderate backscatter.

Compared with the hummocky terrain of the northern segment, the southern segment of the MCSC comprises several smooth‐floored basins, with moderate acoustic backscatter, cut by a series of curvilinear ridges and scarps (bright backscatter ribbons) that generally trend to the NW‐SE (Figures 1, 2, 3). Seismic imaging and sampling by dredging show these are filled with volcanic products (Hayman et al., 2011; van Avendonk et al., 2017). What we refer to here as the “Central Basin” is divided into two oval‐shaped subbasins each 8‐ to 10‐km long and 3‐ to 5‐km wide (Figure 3). These have flat and smooth (volcanic) seafloor and the moderate intensity and homogeneous acoustic backscatter is indicative of thinly draped, sediment‐covered lavas, likely dominated by sheet flows given the smooth surface of the seafloor (Figure 3c). We do not attribute the flat nature of the seafloor in these regions to sediment blanketing over hummocky lavas because such an effect is not observed elsewhere in the axial deep, the sedimentation rate is very low (Land, 1979), and the two areas are seismically interpreted to be deep volcanic basins rather than, for example, flanks of an axial high.

The two subbasins are separated by an obliquely trending, steep‐sided, curvilinear ridge that crosscuts the southern edge of Mount Dent (Figure 3b). While this ridge has been proposed to be an AVR that has propagated into the southern flank of Mount Dent by Cheadle et al. (2012), its steep flanks, sharp curvilinear spine with high acoustic backscatter, and lack of hummocky morphology (Figures 3b and 3c) are evidence of a tectonic origin such as a fault‐bound horst. Where this horst intersects the southern flank of Mount Dent, it continues up‐slope as a deep V‐shaped gully dissecting the massif (Figures 3a and 3b).

**Figure 4:**
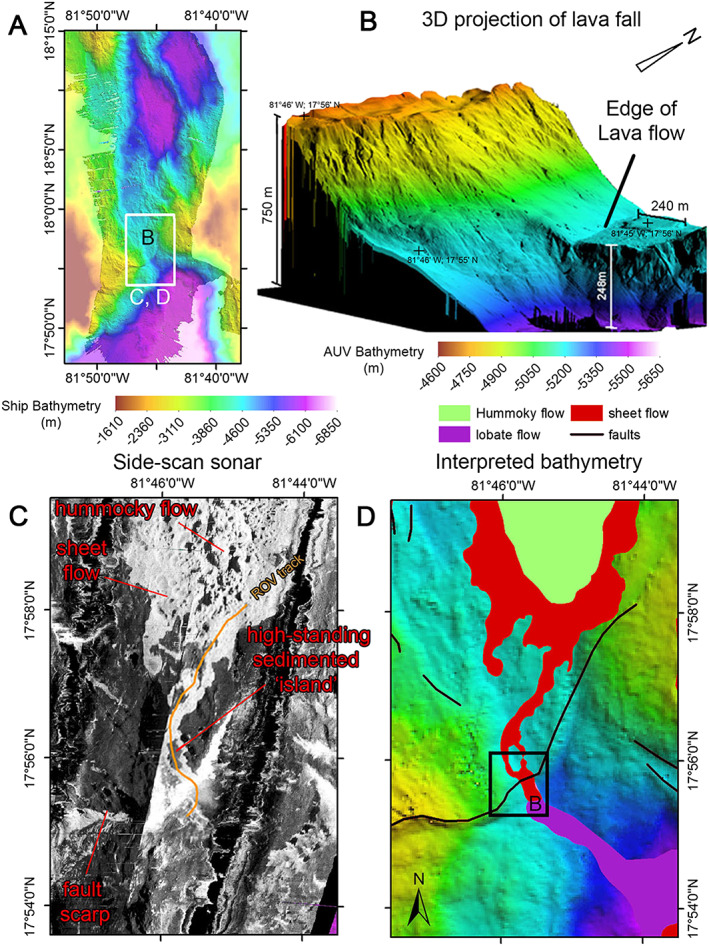
(a) Location of the southern ridge‐transform‐intersection (RTI) between the MCSC and the Swan Island fracture zone, inset box indicates location of panels (c) and (d). Box B is the location of 3‐D image for panel (b). (b) Three‐dimensional projection (from the south) of a “lavafall” formed by a sheet flow as it has cascaded over the RTI fault scarp. The autonomous underwater vehicle bathymetry does not have the same depth color scale as the ship multibeam in panels (a) and (d). (c) Side‐scan sonar image with lava flows appearing as a strong reflector (bright) and sediment covered areas with lower reflectivity. The RTI fault scarp is a very bright curvilinear reflector. Note the presence of a small high‐standing “islands” of sediment covered seafloor surrounded by brighter sheet flows near to and NW of the RTI fault scarp, crossed by an ROV track (light orange). Also the area of diffuse backscatter at the base, and SE of the RTI scarp. (d) Geological map of the southern RTI showing different volcanic terrains and faults.

The southern end of the MCSC is similar to the northern segment, with bathymetry showing elevated hummocky terrain characterized by bright acoustic backscatter, indicative of sediment‐free volcanic terrain along much of the axial floor (Figures 4a–4c). We interpret the lack of sedimentation to be an indicator of relatively young eruptive units compared with lower reflective seafloor that indicates thicker sediment cover. Side‐scan sonar imagery (Figure 4c) also reveals a brightly reflective but smooth area, surrounding the elevated hummocky terrain. The highly reflective area continues southward as a sinuous (in map view) ribbon. This bright and sinuous feature follows the deepest part of the axial floor of the MCSC southward for over 5 km where it surrounds elevated areas of less reflective (i.e., more sediment‐covered) seafloor (Figure 4c). The smooth morphology of this feature suggests that it is a sheet flow with a long and thin runout. In contrast with the adjacent darker seafloor, the bright acoustic backscatter indicates that it is virtually sediment free and thus a relatively recent eruption. This assumption is confirmed by ground truthing using visual observations from the ROV (see Figure 4c for vehicle track in orange) that reveal the bright ribbon to have a smooth lava surface with sparse sediment cover, whereas the darker and more elevated areas are flat‐topped “islands” of seafloor, with thicker (~1 m) sediment cover, which are surrounded by the (relatively) younger sheet flow lava. The AUV‐derived micro‐bathymetry also shows this sheet flow to be cut by a ~250‐m‐high vertical fault scarp that downthrows the axial floor to the south (3‐D projection facing the northwest in Figure 4b, NE‐SW black line in Figure 4d). Close inspection of the side‐scan sonar imagery (Figure 4c) reveals an area of bright and diffuse backscatter at the base of the scarp and a continuation of the lava flow, albeit with lower reflectivity, toward the southeast, away from the bottom of the scarp, and downslope into the nodal deep basin. Visual observation by the ROV of fresh lava draping the top edge and bottom of this scarp is evidence that it once formed a “lavafall” over which the sheet lava flow once cascaded as it flowed south into the nodal basin at the intersection with the Swan Island Fracture Zone. The AUV bathymetry shows the scarp strikes across the axial valley in a SW‐NE orientation as a fault and, from the geometry of its trace as it intersects the topography, has a dip of 62° to the southeast. As such, it marks the intersection between the southern end of the axis of the MCSC and the Swan Island Fracture Zone (Figure 4d).

### 4.2 Geology of the Mount Dent OCC

#### 4.2.1 Overview

**Figure 5:**
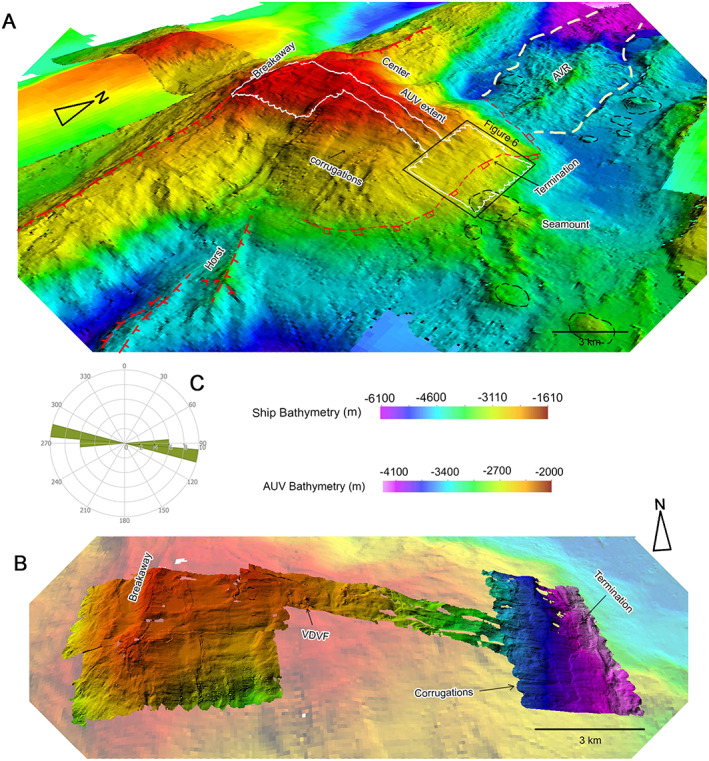
(a) The 50‐m‐resolution 3‐D bathymetric projection of Mount Dent, looking northwest. The extent of the autonomous underwater vehicle (AUV) autosub6000 bathymetry survey is highlighted in white. Black box is the extent of Figure 6. Also outlined in white dashes is the northern axial volcanic ridge, in black dashes are volcanoes, and in red lines are the termination zone (squares on hanging wall) and normal faults (ticks on down‐thrown side). (b) The 2‐m‐gridded resolution AUV bathymetry of the domed surface of Mount Dent, viewed from the south. Note that the AUV and ship bathymetry have different scales. (c) Rose diagram of corrugation orientations.

In contrast with the northern and southern segments of the MCSC, the central segment is dominated by the ~16‐km‐long (E‐W), ~14.5‐km‐wide Mount Dent massif, that rises up to 2,000 m above the adjacent ~4,800‐m‐deep axial floor of the MCSC (Figure 5a). Its smooth surface is cut by N‐S striking faults forming scarps and incisions that strike across the massif (Figure 5a). In E‐W profile, the domed and smooth surface of Mount Dent decreases in its maximum curvature from 8°/km at the base of its eastern flank, where it dips 23° to the east, to 0.2°/km at its summit, where it is nearly horizontal (Figure 5a).

The geology of Mount Dent has been determined from early dredging efforts, Alvin dives, and more recent ROV investigations (Hayman et al., 2011; Stroup & Fox, 1981). Lithologies recovered from the domed surface of Mount Dent include serpentinized harzburgite and dunite, deformed gabbro (including mylonitized and amphibolite‐facies metagabbros), fresh dolerite dikes, and lavas. The western limit of the massif is marked by a 25‐km‐long, N‐S trending ridge with a series of parallel scarps along its crest and orthogonal rills incising its eastern slope (Figure 5a). The smooth, lower southern flank of the massif has a series of E‐W striking, subparallel corrugations (Figures 5a and 5b). Immediately to the east of Mount Dent, the axial floor of the MCSC is characterized by hummocky terrain and circular volcanic edifices. The most prominent of these is a ~2‐km‐diameter volcanic seamount, located 1.5 km east of the base of Mount Dent (Figure 5a). The junction between the hummocky volcanic terrain and smooth lower eastern flank of Mount Dent is marked by a curvilinear fault scarp that trends north‐south and bends around the base of the massif (Figures 5a and 5b).

We recognize the curvilinear scarp separating the smooth lower eastern flank of Mount Dent from the hummocky seafloor to the east as marking the location of the eastward dipping detachment fault that exhumes deep crustal and upper‐mantle lithologies and displaces the neovolcanic hanging wall toward the MCSC axial floor. Tucholke et al. (1998) refer to similar features on the MAR as the *termination*. However, since the geological structures that we map here have a finite width, we adopt the term *termination zone* for convenience. In turn, we define the N‐S elongated ridge, forming the western limit of the Mount Dent massif, as the site of initiation of detachment faulting. Again, following the terminology coined by Tucholke et al. (1998) to describe oceanic detachment faults we refer to this structure as the *breakaway*. Note that the term breakaway generally refers to any region where a fault initially breaks the Earth's surface, and has been widely used to describe continental core complexes (e.g., Davis, 1980). Similarly, termination describes the downdip limit of a fault and has been widely used in marine geology studies to describe where a detachment fault emerges from the subsurface. The terms *hanging wall cutoff* and *footwall cutoff* have also been invoked for termination and breakaway zones in continental and oceanic core complexes (Allmendinger et al., 1981; Escartin et al., 2017), but we do not adopt that terminology here.

#### 4.2.2 The Termination Zone

**Figure 6:**
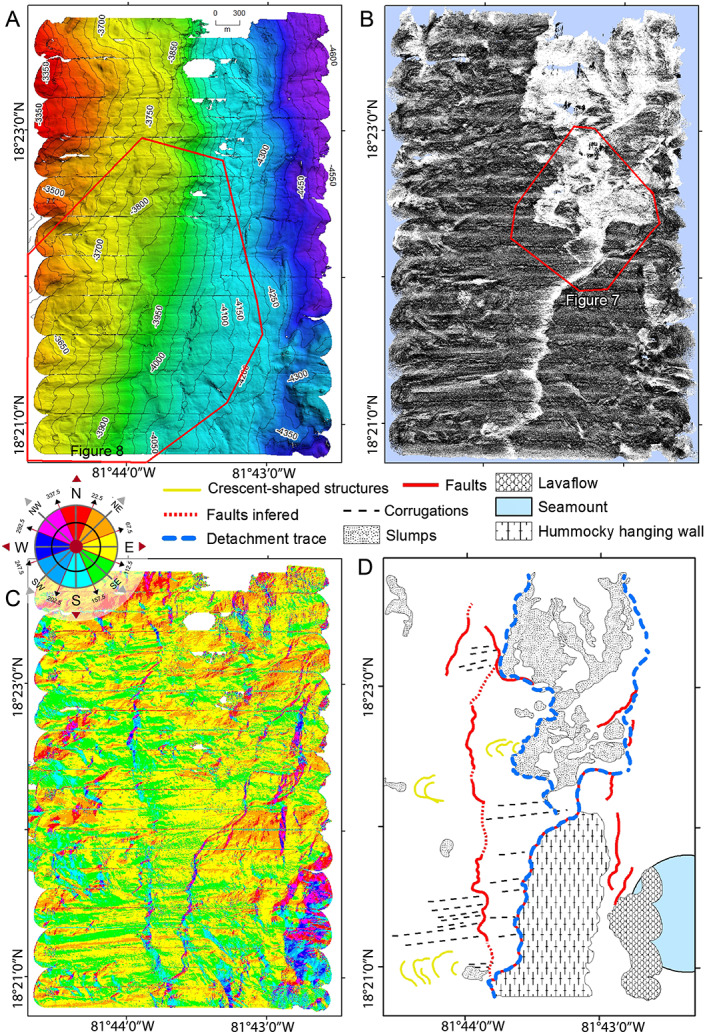
Autonomous underwater vehicle‐derived maps of the lower eastern flank of Mount Dent. (a) The 1‐m resolution, shaded relief bathymetry in the area of the detachment surface indicated in Figure 5. The red outline indicates the extent of Figure 8. (b) The 1‐m‐gridded multibeam backscatter illustrates sedimented areas versus areas with bedrock exposure, based on the latter's stronger (lighter) acoustic reflectivity. The red outline indicates the area of Figure 7. (c) Color‐coded slope azimuth map calculated from the 1‐m‐gridded bathymetry using a 3 × 3 3 Å~ 3 matrix, thereby highlighting fault scarps. (d) Geological interpretation of the termination zone. The dashed blue line is the curvilinear scarp with high backscatter that is interpreted here as marking the trace of the termination zone.

We interpret the history of the termination zone from high‐resolution bathymetry, slope mapping, and acoustic backscatter imagery from the AUV of the base of the smooth, eastward sloping flank of Mount Dent and the hummocky volcanic seafloor of the MCSC axis immediately to the east. In general the area has low acoustic backscatter intensities except for a N‐S trending, curvilinear “ribbon” and chaotic area of high acoustic backscatter (Figures 6a and 6b). The bathymetry and slope‐azimuth map (Figure 6a and 6c) show the bright, curvilinear feature to be associated with a low, west facing scarp that is ~10‐m high at a depth of approximately 4,100 m. High acoustic backscatter is indicative of exposure of hard rock and rough seafloor, whereas lower backscatter reflects sediment cover. High‐intensity acoustic backscatter is found to reduce westward, over a distance of ~100 m, to become similar to the low value of backscatter we find in the surrounding sediment‐covered basement (Figure 6b). We interpret this change in backscatter to reflect increasing acoustic attenuation by a wedge of sediment cover that thickens in the updip direction across the termination zone. This reflects a history of detachment slip in which the accumulation of pelagic sediment progressively attenuates the acoustic backscatter albedo of the detachment surface as it ages from initial exposure immediately adjacent to the hanging wall. We note for context that a virtually identical feature has been imaged (MacLeod et al., 2009) and sampled (Escartin et al., 2017) at the 13°20′N OCC (MAR).

**Figure 7:**
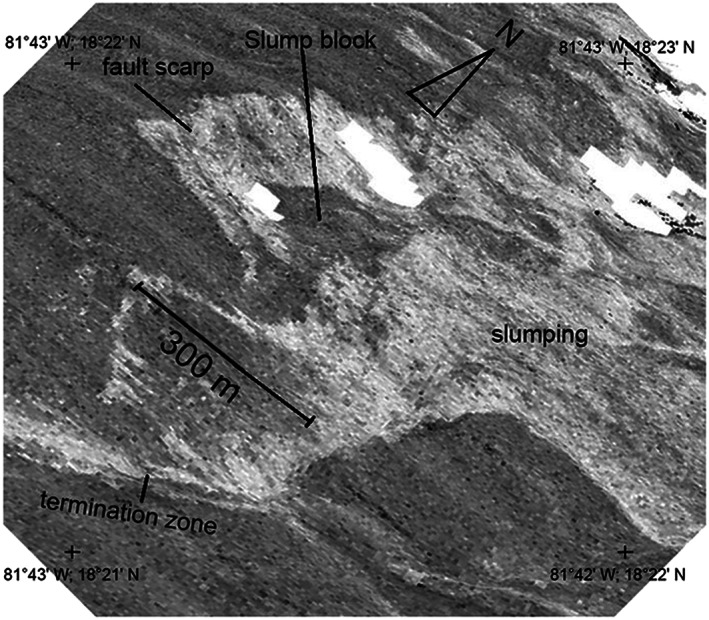
The 3‐D projected autonomous underwater vehicle‐derived multibeam acoustic backscatter of the chaotic region north of where the detachment trace bifurcates, see Figure 6 and georeferenced tick‐marks for location. Consolidated rafts of lower‐backscatter (sediment covered) seafloor surrounded by brighter areas of seafloor indicating freshly exposed harder substrate (slumps) are annotated as a slump block.

The acoustic backscatter image and slope‐azimuth map (Figures 6b and 6c) shows how the westward dipping curvilinear active termination zone merges north of 18°22′N with a broader and more chaotic terrain. The more chaotic terrain comprises patches of low‐backscatter seafloor (i.e., sediment covered), surrounded by a highly reflective (i.e., hard substrate), rugged, and blocky seafloor,. When superimposed on the microbathymetry, the acoustic backscatter image reveals these dark and angular patches to lie within areas of highly reflective and steeply sloping seafloor, characteristic of recent slumping and displacement of “rafts” of sediment‐covered footwall to the east (Figure 7). The microbathymetry and slope‐azimuth maps (Figures 6a and 6c) also reveal another ~10‐m high, N‐S striking, westward facing curvilinear fault scarp that merges with the active termination zone to the south (black line in Figure 6d). The low acoustic backscatter of this scarp shows it to be draped by a continuous sediment blanket, and hence, we infer that it is unlikely to have been recently active.

#### 4.2.3 Corrugations

**Figure 8:**
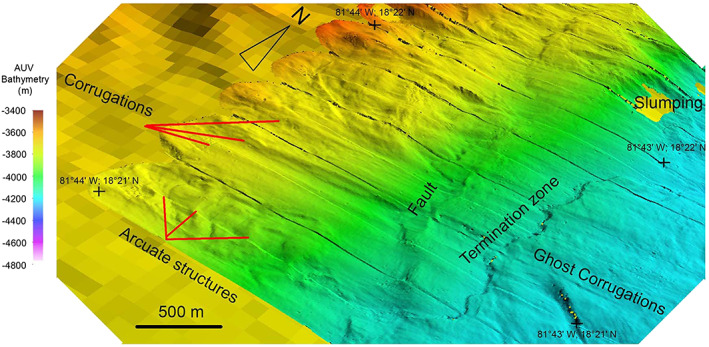
Oblique image of the autonomous underwater vehicle data illustrating corrugations that characterize the lower eastern flank of Mount Dent. See Figure 6a and georeferenced tickmarks for location. See text for discussions of the “ghost corrugations” observable to the east (above) the termination zone and arcuate structures thought to be fault‐related detritus accumulated along the corrugations.

Shipboard multibeam bathymetry data of the flanks of Mount Dent reveal a series of parallel corrugations, undulating grooves observable at the map scale (Figures 5a and 5b;) referred to as *mullion structures* in some areas (e.g., John, 1987). The spatial orientations, described here, were determined via analyses of the bathymetric data. These corrugations trend between 083° and 100° (Figure 5c), approximately parallel to the spreading direction of the MCSC. Smaller‐scale corrugations, observed from the AUV‐derived microbathymetry from near the base of the eastern side of Mount Dent just west of the termination zone, have a slightly different orientation of between 075° and 100°. The variation in corrugation trend at Mount Dent could be due to rheological responses during their evolution as the OCC was exhumed (cf. Escartin et al., 2017). Alternatively, the variation could be due to progressive changes in slip direction and/or the result of deformation of the detachment fault surface after the corrugations formed, such as by folding and/or flexure of the Mount Dent massif. The data also reveal that these corrugations have a wavelength of 100–200 m, an amplitude of 25–35 m, and lengths of up to 1.2 km (Figure 8).

The AUV bathymetry data also reveal arcuate structures superposed on the crests of the corrugations, especially near the termination zone (Figure 8). These arcuate structures are ~10‐m high, up to 1.5‐km long, ~150 m apart, and concave downward toward the east. The origin of these is enigmatic, but we note that they occur to the west of an arcuate bend in the trace of the termination zone (Figure 8) and may result from debris deposited on the footwall by erosion of the hanging wall. We note that similar features are described and sampled from the MAR 13°20′N OCC, by Escartin et al. (2017) and propose that these are common features of oceanic detachment faults where the hanging wall is eroded onto the emerging footwall.

The microbathymetry also reveals similarly oriented corrugations, albeit of a slightly more subdued amplitude, located to the east of the termination zone (Figure 8). These features, termed here *ghost corrugations* are continuous along strike with the corrugations to the west of the termination zone. Their occurrence is enigmatic as they are formed in the rougher terrain of the thin trailing edge of the hanging wall. As such, they are not ornamentations on the detachment surface but might reflect the draping of the thin trailing edge of the hanging wall over topography of the yet‐to‐be‐exhumed footwall (see also MacLeod et al., 2009).

#### 4.2.4 The Upper Slopes of Mount Dent

**Figure 9:**
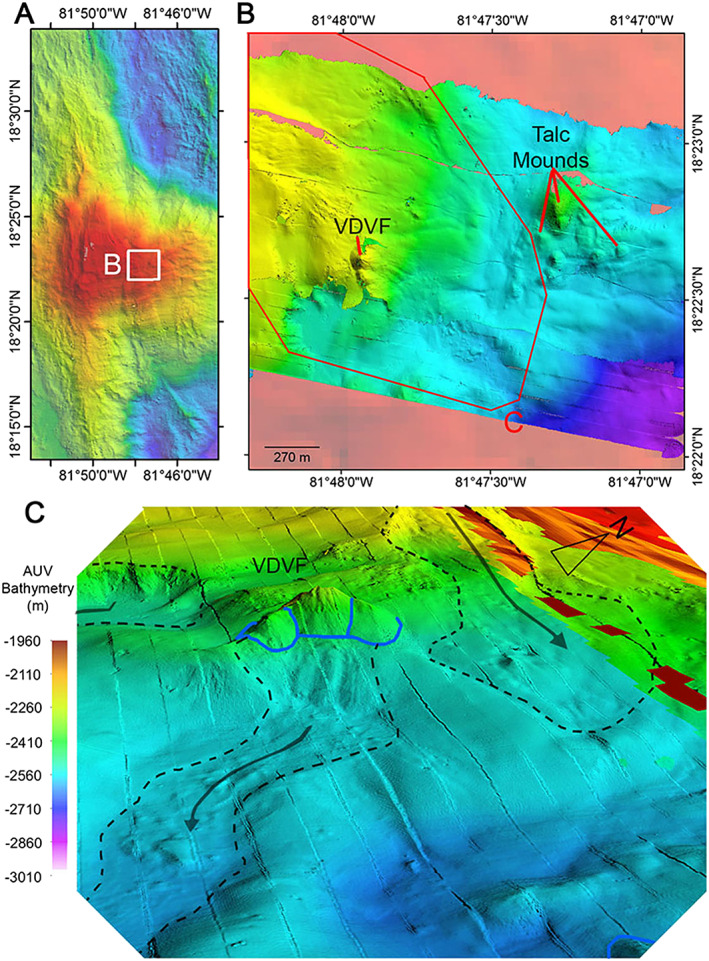
(a) Location map for the Von Damm vent field (VDVF) and talc‐rich mounds on Mount Dent, with the location of panel (b; white square). (b) Autonomous underwater vehicle‐derived 2‐m‐resolution microbathymetry overlain on 50‐m ship‐based bathymetry. (c) The 3‐D projection, looking west, of the VDVF (outlined by the blue lines) showing recent slumping (dashed black lines) and mass wasting (arrows) of material downslope toward the east.

The topography of the upper slopes of Mount Dent, above a depth of 3,000 m, contrast with the smooth eastern and southern flanks of the massif by comprising a blocky and chaotic seafloor with east facing steps and gullies tens of meters deep (Figures 5b and 9). Similar chaotic terrain is reported from the upper slopes of the 13°20′N OCC, MAR, by Bonnemains et al. (2017) and Escartin et al. (2017). Prominent lineaments and scarps in this area are found by ROV observations and sampling to expose gabbro and serpentinized ultramafic rocks (Hodgkinson et al., 2015), and are consistent with faulting of the basement. The most prominent feature in this region is a series of conical mounds, up to 70‐m tall and ~100 m in diameter (Figure 9). These mounds form both the active and inactive vents in the VDVF, located at 81°47′W; 16°22.50′N (Connelly et al., 2012; Hodgkinson et al., 2015). Primary vent fluid, emitted from mounds of hydrothermal talc at 215 °C, has a composition that indicates high‐temperature reaction between seawater, gabbro, and serpentinizing ultramafic rocks (Hodgkinson et al., 2015). The VDVF dissipates up to 500 MW of thermal energy, cooling the interior of Mount Dent (Hodgkinson et al., 2015).

The rocks surrounding the VDVF are mainly gabbroic with some serpentinized ultramafic rocks and relatively fresh diabase dikes, all of which were recovered by ROV as reported in Hodgkinson et al. (2015) and which are consistent with previous sampling studies (Stroup & Fox, 1981). Another prominent but hydrothermally inactive talc mound is located ~500 m to the east of the active VDVF and is surrounded by smaller mounds of a similar origin. These are estimated from the observed thickness of the sediment cover as well as analyses of the vents themselves to have ceased hydrothermal construction at least 20,000 years ago (Hodgkinson et al., 2015), suggesting an extended history of hydrothermal activity at the VDVF.

The active hydrothermal mounds of the VDVF are aligned N‐S and associated with several sets of steep, NNW–SSE trending slopes, interpreted from the AUV‐derived bathymetry, ROV observations, and sampling of gabbroic outcrop as normal fault scarps (Figure 9). In addition, the microbathymetry maps show cusp‐shaped scarps and tongues of disturbed and hummocky material that are elongated downslope toward the east (Figure 9c). This has especially affected the active VDVF mounds, with the eastern slopes showing signs of collapse and mass wasting. To the north of the VDVF, another tongue of blocks and boulders extends to the east and widens into an E‐W trending depression.

#### 4.2.5 The Summit of Mount Dent and Breakaway Zone

**Figure 10:**
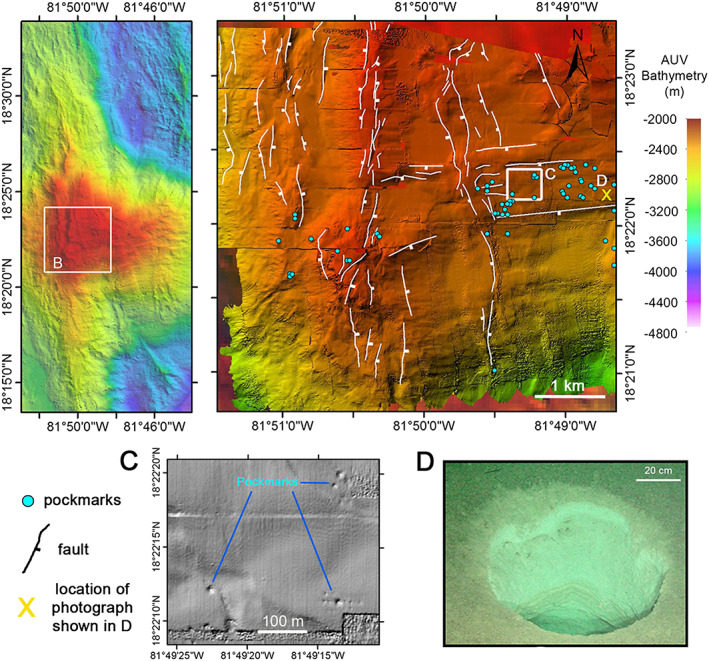
(a) Location map of panel (b), on the crest of Mount Dent. (b) The crest of Mount Dent showing N‐S and E‐W orientated faults scarps (white lines) form grabens which produce basins. Pockmarks (blue dots) identified from autonomous underwater vehicle bathymetry (c). (c) Zoom in of box C in panel (b) showing pockmarks in bathymetry as circular depressions. (d) Photograph from the ROV HyBIS of a pockmark from within the pockmark field located as the yellow “X” in panel (b).

At approximately 81°50′30″W, a N‐S trending (structural) ridge marks the breakaway zone of Mount Dent (Figure 10a). Here, the summit area is characterized by a smooth region of depressions bound between approximately N‐S and E‐W striking ridges and scarps (Figure 10b). These linear features, which have been identified from the high‐resolution multibeam bathymetry data, are likely to be the result of sediment draping over underlying fault scarps.

To the west of the summit ridge, the scarps mainly dip toward the west in a series of steep steps that form the western flank of the breakaway ridge. To the east of the breakaway ridge, the structures dip east and form N‐S elongated basins with smooth seafloor. Despite having only mapped a small area in high‐resolution by AUV, the shipboard multibeam bathymetry map shows the N‐S trending structures continue across the summit of Mount Dent and along the breakaway zone (Figure 10a).

In this area, the AUV‐derived micro‐bathymetry also reveals clusters of pockmarks grouped in an E‐W trending band along the upper southern flank of the summit region (blue dots on Figure 10b and inset detail). The pockmarks are up to 10 m in diameter (Figure 10c). Observations by the ROV of the seafloor in the vicinity of these larger pockmarks (yellow “X” on Figure 10b) reveal steep‐walled circular holes up to 75 cm in diameter and >1‐m deep (Figure 10d). The presence of relatively undisturbed seafloor surrounding the holes is indicative of material having been removed from the subseafloor. These features are characteristic of fluid flow or degassing from the basement as seen in other seafloor environments, albeit in those examples pockmarks are developed in thick sediments (Hovland et al., 2002).

## 5 Discussion

### 5.1 Mount Dent and OCC Evolution

**Figure 11:**
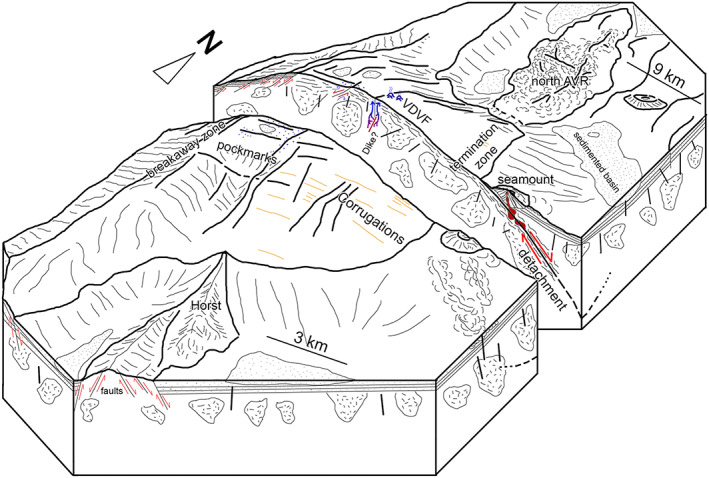
Schematic block diagram illustrating the key elements of the dying stages of the Mount Dent oceanic core complex. The oceanic core complex is formed by detachment faulting, emerging from the termination zone and aging to the west, exposing a footwall that has corrugations parallell to the detachment fault's slip direction, and a heterogeneous “plum‐pudding” crustal structure comprising gabbroic plutons and dikes intruded into a matrix of serpentinized ultramafic mantle material. The northern axial volcanic ridge intersects Mount Dent's northern flank, while in the south, the horst cuts obliquely into Mount Dent's southern flank. The hanging wall immediately to the east and above the detachment fault comprises recent volcanics, including a seamount. Subtle ornamentation depicts hypothesized magmatic intrusions under the Von Damm vent field (VDVF) and the detachment (red) and associated fluid flow (blue).

Mount Dent shares structural similarities with many other OCCs, including a gabbro‐dominated crustal architecture and asymmetric domed surface that dips toward the rift axis where it terminates against hummocky volcanic terrain (Figure 11). Like many of these OCCs, which also host hydrothermal systems, the domed surface of Mount Dent is characterized by corrugations spanning a range of scales from hundreds of meters to kilometers long and tens to hundreds of meters wide and with a narrow range of orientations. Most regional analyses of OCC corrugations find that they trend roughly parallel to the spreading direction (e.g., Smith et al., 2006). Escartin et al. (2017) and Parnell‐Turner et al. (2018), based on their work on the 13°20′N and 13°30′N MAR OCCs, suggested that the corrugations are the product of an integrated evolution of the fault plane as it passes through the brittle‐ductile transition. More generally, corrugated structures on any fault surface can be due to the slip behavior of faults in the brittle crust (Resor & Meer, 2009).

The MAR OCCs in particular exhibit detachment faults that, in places (especially 13°30′N), are cut by high‐angle faults and dike intrusions (MacLeod et al., 2009). MacLeod et al. (2009) proposed that these dike intrusions “kill” detachment faults after they migrate into the axial valley, thereby allowing magmatism from the adjacent AVRs to propagate across the OCC footwalls. Such a model may well apply to Mount Dent which shows evidence of tectonic instability in the: (i) disintegration of its termination zone, (ii) brittle faulting across its upper flanks, summit, and breakaway, (iii) rifting of the southern flank, (iv) the intersection of an axial volcanic ridge with its northern flank, and (v) likely magmatic intrusion deep within the interior of Mount Dent. We suggest that these features are indicative of Mount Dent being in a terminal stage of tectonic seafloor spreading, with slip on the detachment fault virtually ceased, and magmatic spreading reasserting itself as the dominant mechanism of seafloor spreading.

### 5.2 Cessation of Detachment Faulting

Evidence for reduced tectonic activity of the detachment fault comes from near the most recently exposed area of the termination zone where a thin band of acoustic reflectivity rapidly diminishes westward over a distance of ~100 m. We interpret this as a narrow (50–100 m) zone in which a hard and rough basement exposed by slip on the detachment fault gives way to soft sediment cover. Our interpretation is based on the reasoning that at a frequency of 200 kHz pelagic sediment attenuates the backscattered signal from the EM2000 multibeam sonar by ~20–50 dB/m, approximately equal to the difference in acoustic backscatter between hard rock and soft sediment (Mitchell, 1993; Stoll, 1985). A similar observation has been reported for the 13°20′N MAR OCC (e.g., Parnell‐Turner et al., 2018), where a reduction in backscatter intensity across the termination zone is suggested to reflect the changing thickness of sediment covering the fault surface as a result of slip of the detachment fault. There, the surface of the emerging footwall is now covered by basaltic rubble eroded from the hanging wall (Bonnemains et al., 2017).

Although the sedimentation rate for the central MCSC is unknown and is likely to be variable (e.g., Erickson et al., 1972), the regional pelagic accumulation rate has been measured as <5 mm/ka (Land, 1979). We estimate that, given these low sedimentation rates, a reasonable slip rate to cause the observed reduction in acoustic backscatter intensity across the width of the active termination zone would be <5 mm/year. Though just an estimate we suggest that the pattern of sedimentation on the detachment surface indicates that the slip rate is now very slow, if not inactive. In our interpretation, the few millimeters per year deficit between the spreading rate and slip rate is increasingly accommodated by high‐angle faulting and magmatism at this location.

Further evidence that the detachment is currently inactive, or becoming inactive, is seen in the termination zone to the north of 18°22′5″N. Here, the ribbon of bright acoustic backscatter indicates that the active termination zone widens and becomes chaotic. We attribute this backscatter pattern to result from the upper crustal dismemberment of a weak footwall and brittle disintegration of the termination zone. Elsewhere, the west facing curviplanar fault scarp located 1–1.5 km west of the active termination zone (Figure 6) is evidence of vertical dislocation; Stroup and Fox (1981) reported similar fault scarps from their observations from the human‐occupied vehicle Alvin. An alternative interpretation of this particular fault splay is that it is an antithetic normal fault or detachment fault splay formed after the footwall was denuded. In either case, we interpret the bifurcation of faults in the termination zone as evidence of a transfer of stress following strengthening (locking) of the detachment fault due either to its rheologic evolution, rotation to an unfavorable orientation for slip, or both. As the hanging wall and footwall become more tightly coupled, strain is transferred from the low‐angle detachment fault to new and steeper dipping normal faults that cut across the footwall, and the detachment fault surface disintegrates.

The central basin, forming the northern end of the southern segment of the MCSC, is dissected by a NW‐SE trending horst that rifts the southern flank of Mount Dent. Such horsts are components of horst‐and‐graben structures and rift shoulders that are predicted in models of extensional brittle failure of thick lithosphere (e.g., Lavier & Buck, 2002). Similar features have been observed along the axis of the ultraslow spreading Southwest Indian Ridge (Sauter et al., 2013). The intersection between the faults generating the horst and the detachment fault could be a response to a nontransform offset proposed to bound the southern end of Mount Dent (Macdonald & Holcombe, 1978). The horst could also be a result of the generally melt‐poor environment (see below), thereby accommodating tectonic extension and potentially exposing as yet unidentified deeper crustal or upper mantle materials. Alternatively, in some respects the horst bears some resemblance to the area near the Rainbow vent field (MAR 36°14′N) where detachment faulting and magmatic sill emplacement have been identified in a nontransform offset (Eason et al., 2016; Paulatto et al., 2015). Regardless, the fact that the horst is associated with normal faulting along the southern flank of Mount Dent is further evidence that the locus of extension is being transferred away from the detachment fault.

### 5.3 Magmatism and OCC Death

Seismic imaging (van Avendonk et al., 2017), seafloor observations (Hayman et al., 2011), and the new data we present here show that the northern and southern segments of the central MCSC are currently dominated by robust volcanic activity. The northern segment, which our data reveal is typified by a young, hummocky AVR, circular volcanoes, and hydrothermal activity at the Beebe Vent Field, lies directly above a low‐velocity seismic anomaly of probable magmatic origin (van Avendonk et al., 2017). The prominent AVR in this location intersects the northern flank of Mount Dent. Indeed, the presence of a wide field of fresh hummocky volcanics along the northern AVR at its intersection with Mount Dent is evidence for a robust magma supply immediately adjacent to the OCC if not beneath it. Such magmatism would explain the hydrothermal activity at the VDVF near the summit of Mount Dent. Fluid flow is likely facilitated by fracturing and opening of fluid flow pathways over the possible magmatic intrusion and the subsequent mining of heat from deep within the OCC (Hodgkinson et al., 2015; McDermott et al., 2015). The fracturing and magmatism likely contributes further to a low *P* wave seismic anomaly in the deep subsurface below Mount Dent (Harding et al., 2017).

Our data also show that the area between the two volcanic segments of the MCSC, in the hanging wall of the detachment fault, comprises hummocky volcanics including a circular seamount. Seismic studies show this to be underlain by thin crust (Harding et al., 2017; van Avendonk et al., 2017). We note that we cannot rule out that these volcanic features have been tectonically juxtaposed against the footwall of the Mount Dent OCC by the detachment fault. However, if these volcanic features indeed cross cut the detachment fault, then they are further evidence that the OCC is being intruded by magma, contributing to the cessation of detachment faulting.

### 5.4 Hydrothermal Activity and the Mechanical Evolution of OCC Interiors

Following intrusion, Hodgkinson et al. (2015) estimated that the interior of Mount Dent cools by 500 °C over 3800 and 6400 years. This estimate is based on a 10 × 10 × 3‐km volume and a rate of about 0.3 m^3^/s assuming the dominant lithology is gabbro (Arafin & Singh, 2016) and that chemically produced heat from serpentinization is not significant. Such rapid cooling will deepen the brittle‐ductile transition within the OCC footwall, enhancing further brittle deformation, fluid flow, and weakening of the interior of the massif.

Faulting, fracturing and fluid flow are a response to magmatic intrusion within the OCC and also a reflection of the changing stress field as the OCC is flexurally exhumed and spreads off axis. However, the brittle deformation and fluid flow are also likely to change the effective stress within the Mount Dent massif, thereby further weakening the interior of the OCC relative to the detachment fault to the east. Such a strain evolution has also been proposed for the Atlantis Massif, where exhumation is accompanied by flexural uplift and rotation of normal faults into a mechanically favorable high‐angle orientation, accompanied by internal strain within the OCC (Karson et al., 2006). Also at the Atlantis Massif, fluids flow through fractures and faults within the OCC feeding the Lost City Field vents (Denny et al., 2016) and may also cause a change in effective stress due to the overall low‐permeability environment (Hirose & Hayman, 2008). The presence of pockmarks distributed across the southern flank of Mount Dent potentially indicates an even broader fluid flow regime associated with internal deformation of the OCC. We note that the pockmarks themselves do not align along individually mapped faults, illustrating that the permeability and fluid flow regime is more widespread within the faulted regions, perhaps in the distributed fractures of damage zones surrounding the faults themselves.

The idea that the detachment is becoming inactive is also consistent with modeling that indicates that, as conditions become less favorable for continued OCC growth (e.g., through diking and magmatic intrusion across the detachment fault and into the hanging wall), new high‐angle faults begin to dissect the OCC (Behn & Ito, 2008). As discussed above, such a transition may have already occurred at Mount Dent, as suggested by the occurrence of predominately N‐S striking normal faults and recent slumping higher up on the domed massif, the hydrothermal venting, and the clusters of pockmarks indicating fluid released from within the upper massif by brittle faulting. We note that similar tectonic features, other than perhaps the pockmarks, have been reported from OCCs elsewhere, including the FUJI Dome, an inactive OCC on the Southwest Indian Ridge (Searle et al., 2003), and the Rainbow massif on the northern MAR (Paulatto et al., 2015) and suggest this style of faulting is associated with OCC termination.

### 5.5 OCC Evolution at Ultraslow Spreading Centers

Before explaining how our interpretations and hypotheses are consistent with current views of ultraslow spreading centers, we note that there are alternative hypotheses for the origin of several of the key geologic features. Though singularly we cannot rule these hypotheses out, we find them to be less consistent both with our data and with the model framework we describe below. For example, the detachment could be currently slipping at an unchanged rate, but then we would expect more irregular, unsedimented areas of the detachment surface near the termination zone as seen, for example, at the Kane transform OCC of the MAR (Tucholke et al., 2013). Furthermore, if the detachment was still active, we would expect sedimentary wedge and hanging wall normal fault relations more consistent with a Coulomb Wedge model (Hayman et al., 2003; Olive et al., 2019); we observe neither of these features. Magmatic diking could have formed the southern horst, but we observe no neovolcanic zone associated with this feature on its trace along the seafloor. Lastly, lithospheric heat (Lowell, 2017) and/or heat from serpentinization (Früh‐Green et al., 2003) could be driving the hydrothermal system. However, the fluid and mineral chemistry at the VDVF (Hodgkinson et al., 2015; McDermott et al., 2015; Webber et al., 2015), the spatial coincidence with the nearby AVR, the similarities of the setting with the magmatic sills seismically imaged below the possibly analogous Rainbow vent field on the MAR (Canales et al., 2017), and arguments that venting requires magmatic heat (Allen & Seyfried, 2004), all lead us to suspect that there are gabbroic intrusions from the mantle intruding the OCCs.

We now describe how our interpretations and hypotheses are oddly consistent with current views of ultraslow seafloor spreading. Ultraslow spreading centers worldwide are thought to have relatively thin crusts and great axial depths, a reflection of generally low melt production from a mantle with a low potential temperature (e.g., Dick et al., 2003; Klein & Langmuir, 1987). The MCSC falls within this class of ultraslow spreading centers, and seafloor older than 10 Ma is dominated by exhumed mantle (Grevemeyer et al., 2018), and serpentinized peridotites from the axial deep resemble those from the Southwest Indian and Gakkel Ridges (Mallick et al., 2014). Yet, Mount Dent is also similar to OCCs on other ultraslow spreading ridges, such as Atlantis Bank on the Southwest Indian Ridge (SWIR) which despite the overall melt‐poor environment have drill cores dominated by plutonic gabbro bodies intruded into an ultramafic host rock (Dick et al., 2000).

With an E‐W length of 14 km from the termination zone to the breakaway, and a full spreading rate of 15 mm/year, we calculate that the Mount Dent OCC was active for between about 1 and 2 Myr, given that the youngest edge of magnetic anomaly 2 (~1.64 Ma, Leroy et al., 2000) coincides approximately with the breakaway region (Hayman et al., 2011). We note that even though magnetic anomalies are highly asymmetric in their character from west to east across Mount Dent, Anomalies 2 and 3 are roughly symmetrically located on the conjugate sides of the spreading center (Hayman et al., 2011; Leroy et al., 2000; Rosencrantz, 1988). Given that the eastern flank of the central MCSC is dominated by volcanic rocks and the western side by detachment faulting at Mount Dent, the symmetry in spreading indicates that tectonic extension on the OCC detachment fault accommodates ~50% of the plate separation while magmatic accretion accommodates the other 50%.

In the terminology of Buck et al. (2005) the MCSC seafloor spreading is thus described by an *M* (ratio of tectonic to magmatic spreading) of 0.5. Olive et al. (2010) argue that termination of OCC growth is favored where this proportion of magmatism is intruded into the brittle lithosphere deep within the footwall of an OCC. Thus, ironically, even though overall the MCSC is a melt‐poor environment, local magmatism can accommodate enough seafloor spreading so as to disfavor OCC development as is observed at faster spreading centers worldwide.

## 6 Conclusions

Interpretations of multibeam bathymetric and side‐scan sonar data highlight how a range of tectonic and magmatic processes is impacting a prominent OCC, Mount Dent, that defines the central east flank of the ultraslow spreading MCSC. Extensive and recent volcanism in the northern axial valley of the MCSC leads to southward prolongation of an AVR into the northern flank of the OCC. In the southern axial valley an extensional fault system generates a horst that continues northward into the southern flank of the massif. Faulting and distributed fracturing cut across the OCC‐bounding detachment surface leading to significant mass wasting in several locations. Pelagic sedimentation unevenly drapes the corrugated detachment surface, but in a manner that suggests recent detachment exhumation at a slower rate than tectonic spreading. The propagation of magmatism and faulting into the massif's flanks from the north and south allows the transfer of strain from the OCC detachment fault to the steeply dipping normal faults that dissect the flexed massif. Deep‐rooted hydrothermal activity cools the interior of OCCs and deepens the ductile/brittle transition, increasing the volume of footwall that undergoes brittle extensional deformation and further weakening the footwall. Some alternative hypotheses cannot be ruled out, including possible ongoing detachment faulting, significant contributions from other heat sources to the hydrothermal system, and magmatic intrusions associated with the southern horst. However, our favored interpretations are in broad agreement with both existing subsurface geophysical data and geodynamic models for OCC evolution, whereas collectively the alternatives are not. Namely, that models predict that OCC termination occurs when magmatic intrusion and diking into the brittle part of an OCC footwall exceeds 50%. This model prediction, when applied to the MCSC, leads to the conclusion that despite the melt‐starved nature of ultraslow spreading ridges, tectonic spreading by OCC growth is terminated by an increase in magmatic activity, as is observed for faster spreading centers.

## Supporting information

Supporting Information S1

Figure S1

Figure S2

Figure S3
