# Supplementary material for: Volcanic‐Tectonic Structure of the Mount Dent Oceanic Core Complex in the Ultraslow Mid‐Cayman Spreading Center Determined From Detailed Seafloor Investigation
Source: Geochem Geophys Geosyst. 2019 Mar 7;20(3):1298–318. doi: 10.1029/2018GC008032 (PMC9285398; doi:10.1029/2018GC008032)
Supplement: Supplementary file 1 — Supporting Information S1 [file GGGE-20--s001.docx]

*Geochemistry, Geophysics, Geosystems*

Supplemental Information for

**Volcanic-tectonic structure of the Mt. Dent Oceanic Core Complex in the ultraslow Mid-Cayman Spreading Center determined from detailed seafloor investigation**

**G. Haughton^1^, N. W. Hayman^2^, R. C. Searle^3^, Tim Le Bas^4^, and B. J. Murton^4^**

^1^School of Ocean and Earth Sciences, ^4^National Oceanography Center, University of Southampton Waterfront Campus, European Way, Southampton, SO14 3ZH, UK, ^2^University of Texas, Institute for Geophysics, Jackson School for Geosciences, 10100 Burnet Rd., Austin TX, 78758, USA, ^3^Durham University, Department of Earth Sciences. Durham, DH1 3LE, UK, ^4^National Oceanography Center, European Way, Southampton, SO14 3ZH, UK.

**Contents of this file**

Figure S1 to S3

**Introduction**

This supporting information provides the three files containing the bathymetric and sidescan sonar data used in the manuscript. All three are tailored to a GIS or QGIS system. Geographic coordinate systems, key coordinates, resolution, and bit size are as follows:

Supplemental Material 1 - GeoTiff file from shipboard multibeam system gridded at 50m in WGS84 coordinates. Top Left = 18.8291N/82.0299W, Bottom Right = 17.5986/81.3338W, 1537 X 2717 pixels, 32 Bit

Supplemental Material 2 - GeoTiff from 30kHz Sidescan Sonar TOBI gridded at 12m, in UTM Zone 17N WGS84, 2387 X 8310 pixels, 8 Bit

Supplemental Material 3 - GeoTiff file from Autosub6000 multibeam system, gridded at 3m in UTM Zone 17N WGS84. 5747 X 2164 pixels, 32 Bit
