# Supplementary figures and images for: Volcanic‐Tectonic Structure of the Mount Dent Oceanic Core Complex in the Ultraslow Mid‐Cayman Spreading Center Determined From Detailed Seafloor Investigation
Source: Geochem Geophys Geosyst. 2019 Mar 7;20(3):1298–318. doi: 10.1029/2018GC008032 (PMC9285398; doi:10.1029/2018GC008032)

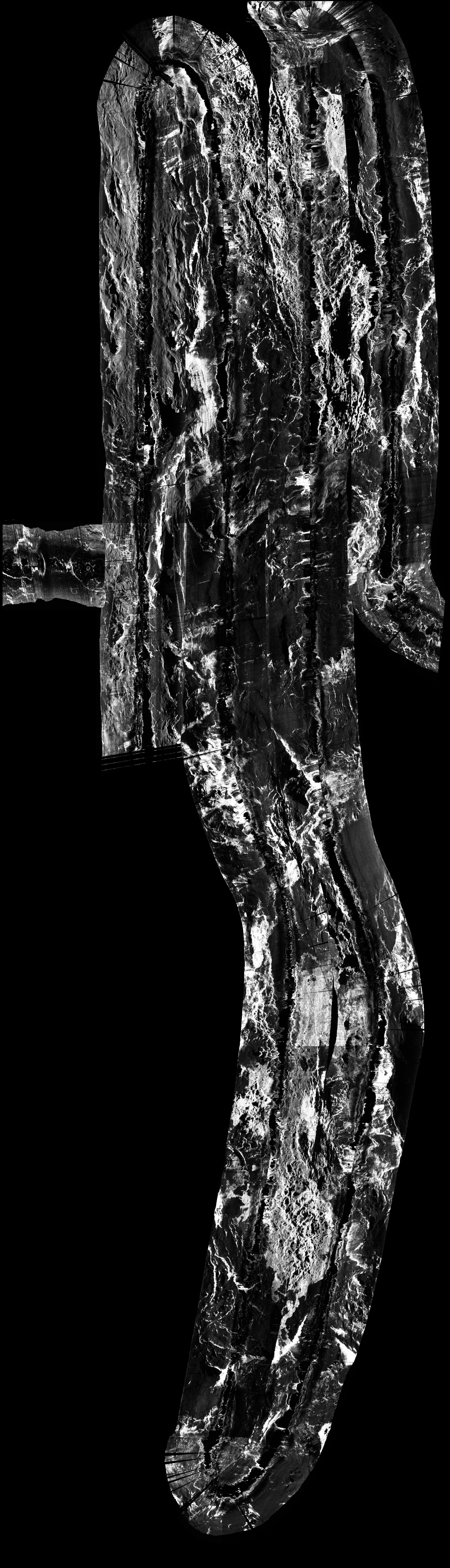

Supplement: Supplementary file 3 — Figure S2 [file GGGE-20--s003.tif]
